# Conditioned medium of engineering macrophages combined with soluble microneedles promote diabetic wound healing

**DOI:** 10.1371/journal.pone.0316398

**Published:** 2025-03-12

**Authors:** HongYu Wang, BaoHua Wei, Hasi WuLan, Bin Qu, HuiLong Li, Jing Ren, Yan Han, LingLi Guo

**Affiliations:** 1 Medical School of Chinese PLA, Department of Plastic and Reconstructive Surgery, First Medical Center of Chinese PLA General Hospital, Beijing, China; 2 Department of Burn and Plastic Surgery, PLA No.983 Hospital, Tianjin, China; 3 Department of Burn Surgery, Sichuan Academy of Medical Sciences & Sichuan Provincial People’s Hospital, Chengdu, China; 4 College of Basic Medical Sciences, School of Medicine, Zhejiang University, Hangzhou, China; Jinan University, CHINA

## Abstract

Diabetic wounds have a profound effect on both the physical and psychological health of patients, highlighting the urgent necessity for novel treatment strategies and materials. Macrophages are vital contributors to tissue repair mechanisms. Macrophage conditioned medium contains various proteins and cytokines related to wound healing, indicating its potential to improve recovery from diabetic wound. Engineering macrophages may enable a further improvement in their tissue repair capacity. Fibroblast growth factor 2 (FGF2) is a crucial growth factor that plays an integral role in wound healing process. And in this study, a stable macrophage cell line (engineered macrophages) overexpressing FGF2 was successfully established by engineering modification of macrophages. Proteomic analysis indicated that conditioned medium derived from FGF2 overexpressed macrophages may promote wound healing by enhancing the level of vascularization. Additionally, cellular assays demonstrated that this conditioned medium promotes endothelial cell migration in vitro. For the convenience of drug delivery and wound application, we prepared soluble hyaluronic acid microneedles to load the conditioned medium. These soluble microneedles exhibited excellent mechanical properties and biocompatibility while effectively releasing their contents *in vivo*. The microneedles significantly accelerated wound healing, leading to a marked increase in vascular proliferation and improved collagen deposition within a full thickness skin defect diabetic mouse model. In summary, we developed a type of hyaluronic acid microneedle loaded with conditioned medium of engineered macrophages. These microneedles have been demonstrated to enhance tissue vascularization and facilitate diabetic wound healing. This might potentially serve as a highly promising therapeutic approach for diabetic wounds.

## Introduction

Diabetic wound represent one of the most prevalent challenges in the field of wound repair [1]. Up to now, there has been no satisfactorily effective method for the clinical treatment for diabetic wounds, which brings great economic burden to society and patients [2,3]. According to projections from the International Diabetes Federation (IDF), the number of individuals with diabetes in China will reach 174.4 million by 2045. Among them, at least 5%-10% of diabetic patients will face diabetic wound problems [4]. This issue not only constitutes a critical social problem that cannot be ignored, but also represents an urgent scientific challenge that necessitates resolution within the medical field. The elevation of blood glucose in diabetic patients leads to peripheral nerve damage, microcirculation disturbance, and local infection. Diabetic wounds are marked by persistent inflammatory response, insufficient tissue perfusion, local wound deficiencies and protracted healing times [5]. Owing to the complexity of the microenvironment of diabetic wounds, the outcomes of wound treatment are frequently unsatisfactory. To enhance the therapeutic effect of diabetic wounds, we explored and developed a novel soluble hyaluronic acid microneedle dressing loaded with the conditioned medium of engineered macrophages.

Previous studies have shown that the conditioned medium of macrophages possesses the potential for tissue repair. Macrophages are widely recognized as pivotal regulators in the wound healing process, playing a critical role in wound immune regulation, inflammation and tissue regeneration [6,7]. The conditioned medium derived from macrophages contains a diverse array of bioactive substances, including extracellular vesicles, exosomes, proteins, nucleic acids, lipids and so on. These key bioactive substances often play significant biological roles in tissue repair. A study has indicated that potential therapeutic targets for treating diseases or injuries of the central nervous system might exist in macrophage conditioned medium [8]. Li found that CXCL-10/CXCR3 mediated tissue repair by modulating the expression of arginine-1, vascular endothelial growth factor-a (VEGF-a) and TNF-α in macrophages [9]. Furthermore, fibroblasts exposed to macrophage conditioned medium exhibited phenotypic changes and alterations in collagen organization [10]. Basu’s study showed that conditioned medium derived from macrophages incubated with platelet derived growth factor (PDGF) and a combination of VEGF+PDGF under inflammatory conditions enhanced phagocytic activity in olfactory ensheathing cells [11]. However, there are few studies focus on the effects of macrophages conditioned medium on wound healing. Based on previous studies, we speculated that conditioned medium of macrophages might be a prospective therapeutic method for diabetic wounds.

To further enhance these abilities to promote wound healing, we have also carried out engineering transformation on macrophages. Engineered macrophages refer to the modification of macrophages through genetic engineering or cell engineering techniques to enhance their functions or alter their characteristics for specific therapeutic or research purposes. Engineered macrophages possess extensive application prospects in medical research and clinical therapeutics. Fibroblast growth factor 2 (FGF2) is an important angiogenic factor that stimulates the proliferation of various cells types, including vascular endothelial cells and fibroblasts [12]. It has been shown to induce the migration and chemotaxis in vascular endothelial cells, exhibiting potent pro-angiogenic properties. It is an important cytokine to improve the treatment of ischemic wounds. Researchers found that FGF2 significantly accelerated the closure of refractory burn wounds in diabetic mice [13]. Our previous study also proved that FGF2 could promote the healing of ischemic wound in rabbit ear [14]. Therefore, we endeavored to establish a stable macrophage cell line with overexpression of FGF2, in an attempt to further enhance their tissue repair capacity.

In order to improve the administration efficiency, we chose soluble hyaluronic acid microneedles as the drug delivery carrier. Soluble hyaluronic acid microneedles were a delivery carrier that could penetrate the skin and quickly release drugs. Compared with other delivery methods, microneedles have emerged as a focal point in wound management research due to their high drug loading and delivery capabilities, minimal invasiveness, convenience, and strong adhesion properties, which collectively reduce patient discomfort and enhance compliance [15,16]. In recent years, there were many researches on the preparation of various types of microneedles [17,18]. As an effective drug carrier, it could deliver drugs directly into tissues which were beneficial to obtain better treatment effect.

So far, there have been limited studies on the effect of conditioned culture medium of engineering macrophages on diabetic wounds. Consequently, the objective of this study was to establish a cell line of FGF2 overexpressed macrophages and to obtain its conditioned medium. The impact of macrophages conditioned medium on wound healing was explored. The conditioned medium was combined with soluble hyaluronic acid microneedles to enhance its drug delivery efficiency and evaluate its effect in promoting wound repair *in vivo*. This study aims to develop a novel composite microneedle dressing that could enhance tissue vascularization and promote wound healing in diabetic patients. Especially, these will provide a novel strategy for diabetic wound repair through the concept of “engineering macrophages as the pharmaceutical factories” (Scheme 1).

## Method

### Cell culture and transfection

RAW 264.7 cells (Servicebio) were cultured in a 37.5 °C incubator with 5% CO_2_ using Dulbecco’s Modified Eagle Medium (DMEM, Eallbio) containing 10% fetal bovine serum (Gibco) and 1% penicillin and streptomycin (Beyotime). The transfer vector plasmid (pCDH-CMV-MCS-EF1-CopGFP-T2A-Puro-Fgf2), which encodes FGF2 gene along with a green fluorescent protein (GFP) tag, was synthesized and subsequently expressed in RAW 264.7 cells via lentiviral transduction.

### Conditioned medium collection

The transfected RAW 264.7 cells (FGF2 overexpressed group) and RAW 264.7 cells (control group) were cultured respectively in DMEM without fetal bovine serum (FBS) and antibiotics for 24 hours. The cell supernatant was then collected by centrifugation (1000 revolutions per minute for 3 min, followed by 3000 revolutions per minute for 10 min).

### Fluorescence staining

The prepared FGF2 overexpressed RAW 264.7 cells were embedded in paraffin wax and treated with the primary antibody (fgf2-rfp, Servicebio, 1:1000). The sections were placed flat in a humidified chamber at 4 °C and incubated overnight. Then, the slides were washed three times for 5 minutes each in PBS (PH 7.4) using a decolorizing shaker. The secondary antibody (CY3, Servicebio, 1:300) was then applied and incubated at room temperature for 50 minutes in the dark. Afterward, the slides were rinsed three times for 5 minutes each in PBS (PH 7.4) while shaking on the decolorizing shaker. Finally, DAPI (4’,6-diamidino-2-phenylindole) dye solution was added and incubated for 10 min at room temperature under the condition of avoiding light. Images were captured using a positive fluorescence microscope (Nikon Eclipse C1) and a scanner (3DHISTECH Pannoramic MIDI). The excitation wavelength of DAPI was set to range from 330 to 380 nm with an emission wavelength of 420 nm. The corresponding conditions for CY3 were 510 to 560 nm and an emission wavelength of 590 nm.

### Enzyme-Linked Immunosorbent Assay

The Enzyme-Linked Immunosorbent Assay (ELISA) was performed according to the manufacturer’s protocol (Elabscience, E-EL-M0170). A volume of 100 μL of standards or samples was added to each well of 96-well plate and incubated at 37 °C for 90 minutes, with three wells designated for each sample. Subsequently, biotinylated antibody, streptavidin–horseradish peroxidase reagent and 3, 3′, 5, 5′-Tetramethylbenzidine substrate were introduced into the wells in accordance with the established protocol. The plate was developed at 37 °C without light until an obvious blue gradient. Thereafter, a stop solution was added to each well. The optical density (OD) of each well was measured at 450 nm by enzyme-labeled instrument. A standard curve was constructed to analyze the corresponding concentration.

### Real-time quantitative PCR

The primers utilized for mRNA detection were synthesized by Servicebio Biotech, and the sequences of the primers are presented in Table 1. Thermal cycling was performed using QuantStudio™ 3 Real-Time PCR System (Applied Biosystems) under the following conditions: denaturation at 94 °C for 30 seconds, followed by 45 cycles of amplification consisting of denaturation at 94 °C for 5 seconds and annealing/extension at 60 °C for 30 seconds.

**Table 1:** Primer used for RT-qPCR.

Primer	Sequence (5’ to 3’)
NM_008006.2 Mouse-bFGF	Forward: GAGCGACCCACACGTCAAAC
	Reverse: CAGCCGTCCATCTTCCTTCATA
NM_007393.3 Mouse-β-actin	Forward: GTGACGTTGACATCCGTAAAGA
	Reverse: GTAACAGTCCGCCTAGAAGCAC

### Proteomics

Protein analysis was performed using a label-free quantitative technique based on mass spectrometry. The conditioned medium (200 μL) was transferred to a 3 kD ultrafiltration tube and centrifuged at 12000 relative centrifuge force at 4 °C for 10 minutes. This step was repeated three times. The samples were subsequently subjected to SDS-PAGE electrophoresis (80 V for 15minutes, followed by 120 V for 70 minutes). Then, the pre-machine treatment steps such as reduction alkylation, acetone precipitation, solution digestion (Trypsinn enzyme, 0.25 μg/μL, incubated in a constant temperature incubator at 37 °C for 16 hours) and C18 desalination were performed. Analysis was carried out using a liquid chromatograph-mass spectrometer (Vanquish/Orbitrap Fusion™ Lumos™ Tribrid™, Thermo Fisher Scientific). The full scanning range of mass spectrum was 300–1800 m/z. Primary mass spectrum resolution was configured to be 120000 (AGC: 4e5, MaximumIT: 20 ms). Secondary mass spectrum resolution was set to be 15000 (AGC: 5e4, MaximumIT: 22 ms). And the peptide fragmentation collision energy was established at 30%. Finally, raw data (.raw) from the mass spectrometry analysis were generated. MaxQuant software was employed to search the target protein database for the original mass spectrometry file with the following parameters: (1) Fixed modifications: Carbamidomethyl (C); (2) Variable modifications: Oxidation (M), Acetyl (Peptide N-term); (3) Enzyme: Trypsin; (4) Database: Uniprot_Mus; (5) Maximum Missed Cleavages: 2; (6) Primary Mass Tolerance (Peptide Mass Tolerance): 20 ppm; (7) Secondary Mass Tolerance (Fragment Mass Tolerance): 20 ppm. Filtration condition had been set for protein: Unique peptide ≥ 1.

### Preparation and characterization of hyaluronic acid microneedles

Sodium hyaluronate powder was dissolved in purified water or macrophage conditioned medium for swelling in a water bath. After the bubbles were removed at room temperature, a clarified solution of hyaluronic acid was obtained. The clarified sodium hyaluronate solution was injected into the Polydimethylsiloxane (PDMS) microneedle mold and shaken back and forth to ensure even coating. Sodium hyaluronate solution was introduced into the needle cavities of the PDMS mold using vacuum negative pressure defoaming techniques. The mold was dried in an oven for 6 hours until the microneedles could be released, and then hyaluronic acid microneedles were obtained. The morphology of microneedles was examined using scanning electron microscope (SEM, ZEISS EVO25).

Microneedle indentation: The microneedle was inserted into the skin tissue of the mouse’s back and removed after 5 minutes. Subsequently, the skin tissue was excised for paraffin embedding, sectioning and observation (Shengqiang Technology Co., Ltd., SQS-40p).

Mechanical property test *in vitro*: The mechanical stress of the prepared microneedles was tested using a texture analyzer. The microneedle patch was positioned horizontally on the platform with the needle tips facing upwards. A probe compressed the microneedles vertically at a rate of 0.1 mm/s, and the corresponding compression force was recorded.

Dissolution and release property *in vitro*: FGF2 was used to simulate drug release *in vitro*. A total of 1 mg of FGF2 was dissolved in 1 mL of sterile deionized water, and 300 μL was added into 5.7 mL of sterile deionized water. After thorough mixing, 300 mg of small molecule hyaluronic acid was incorporated to prepare a solution with concentrations of 50 μg/mL for FGF2 and 50 mg/mL for small molecule hyaluronic acid. Following complete dissolution using a magnetic stirrer, the drug solution was injected into the PDMS mold with a pipette gun for vacuum negative pressure defoaming. After drying and stripping, the production of physically embedded FGF2 microneedles is completed. The prepared microneedles were then placed in 30 mL of sterile deionized water, and then placed under a constant temperature shaking table for release (150 r/min, at 37 °C). During the release process, samples were collected at intervals of 1 min, 2.5 min, 5 min, 7.5 min, 10 min, 15 min, 20 min, 30 min, 40 min, 50 min and 60 min respectively. The samples were diluted separately and the FGF2 concentration in the samples was detected by Elisa kit. The absorbance of the sample was measured by enzyme-labeled apparatus. FGF2 release concentration was obtained from measured data and standard curve.

Cytocompatibility test: The microneedles were immersed in the medium to obtain the tip soaking solution. Human umbilical vein endothelial cells (HUVECs) were then seeded into a 96-well plate to allow for cell attachment. FGF2 solution, blank microneedle soaking solution and FGF2 overexpressed macrophage culture medium microneedle soaking solution were used to replace the culture medium respectively. After incubation for 24 h, Cell Counting Kit-8 (CCK-8) Assay Kit was used to assay cytotoxicity *in vitro*.

### Cell scratch assay

The conditioned medium was utilized for the *in vitro* cell scratch assay. EA.hy 926 cells in exponential growth were seeded into 6-well plates at a density of 5 × 10^5^ cells per well and incubated for 24 hours. Once the cells reached approximately 90% confluency, a scratch was created using a 100 μL pipette tip to assess cell migration. Following the addition of conditioned medium from the transfected macrophages to the EA.hy 926 cells, culture was continued for an additional 24 hours. Cell migration was observed using a microscope (Nikon, FHEIPSE Ti) at various time intervals. The relative area of scratch closure was calculated by determining the cell-free gap area using ImageJ software. This calculation employed the following equation: relative area of scratch closure (%) =  (initial wound area - wound area at time t)/ initial wound area ×  100. PBS solution was administered to the control groups.

### Diabetic mouse wound model

To induce type 2 diabetic mouse model, male Balb/c mice (6 weeks old) were fed a high-fat diet for 4 weeks, followed by a 24-h fast period and intraperitoneal injections of streptozotocin (STZ) at a dosage of 40 mg/kg. One week later, blood was collected via the tail vein to measure the blood glucose concentration, and the urine glucose levels was assessed. Mice with blood glucose levels below 16.7 mmol/L were excluded from the study. Following this, an intraperitoneal injection of pentobarbital sodium (3%, 20 mg/kg) was administered. A round wound with a diameter of 1.0 cm was then created on the back of each mouse, extending down to the myofascial membrane to establish a skin wound model of diabetic mice.

### Ethics statement

The Ethics Committee on Animals of the Institute of Radiological Medicine of the Chinese Academy of Medical Sciences ensured that all animal experiments were conducted in accordance with their guidelines (approval number: IRM/2-IACUC-2404-014). Each mouse was provided with ad libitum access to water and food throughout the experiment and was euthanized via CO_2_ inhalation. Every effort was made to minimize distress of the mice.

### Wound healing assay *in vivo*

A total of 20 diabetic mice were randomly assigned to four groups: Control group, microneedles group (MN group), conditioned medium +  microneedles group (CM +  MN group) and FGF2 overexpression CM +  MN group (FGF2 OE CM +  MN group). The control group received treatment with sterile gauze, while the MN group was treated with microneedles without conditioned medium. The CM +  MN group underwent microneedle therapy containing macrophages conditioned medium. In the FGF2 OE CM +  MN group, microneedles infused with conditioned medium from FGF2 overexpressed macrophages were utilized for wound treatment. All dressings were secured in place using tape (3M).

Regular dressing changes was performed on the wounds, and the healing process was observed and documented via camera over a period of 12 days. The rate of wound healing was evaluated using the following equation: relative wound area =  (wound area on day 0 −  wound area on day t)/ wound area on day 0.

### Histologic and immunohistochemical analysis

A sample of skin was collected from the wound site of the sacrificed mice and fixed with 4% paraformaldehyde. Subsequently, the samples were dehydrated and embedded in paraffin. The paraffin was sliced (Leica RM2245, 6 μm) and then stained with hematoxylin-eosin (H&E) and Masson’s trichrome. To assess angiogenesis during the healing process, immunohistochemical staining was conducted on the sections. This technique was employed to visualize platelet endothelial cell adhesion molecule-1 (CD31) (brown-yellow, DAB color development kit, Beijing Zhongshan Jinqiao Biotechnology Co., LTD), which is the marker of endothelial cells. The results were examined using a microscope (OLYMPUS, DP26).

### Statistical analysis

The data were presented as the mean±SD. All statistical analyses were carried out and graphs were generated using GraphPad Prism 10.0 and Adobe Illustrator software. Student’s t test was employed to undertake comparisons between two groups. For comparisons among multiple groups, one-way analysis of variance was utilized. Significant differences are indicated by asterisks (*P <  0.05, **P <  0.01, ***P <  0.001, ****P <  0.0001).

## Results and discussion

### Establishment of FGF2 overexpressed macrophages cell line

**Fig 1:**
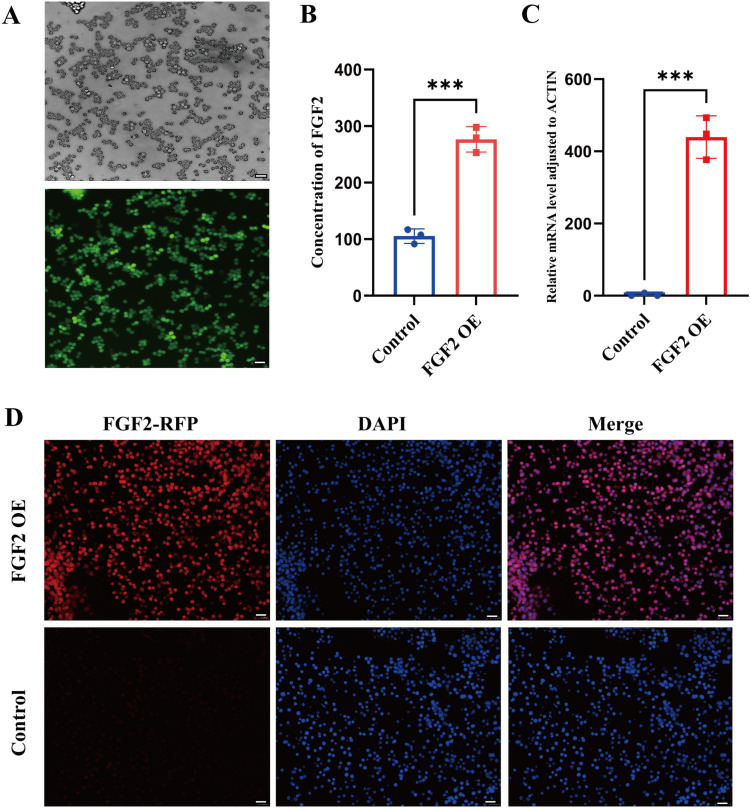
Establishment of FGF2 overexpressed macrophages cell line. A) The green fluorescence in macrophages after transfection. B) ELISA results of the content of FGF2 in macrophages conditioned medium after transfection. C) The mRNA expression of FGF2 gene was measured via RT-qPCR after transfection. D) RFP was used to label FGF2 in RAW 264.7 cells, and the red fluorescence was observed in FGF2 OE group. Data are presented as mean ±  SD. * p <  0.05, **p <  0.01, ****p <  0.0001; OE overexpression; scale bar =  20 μm.

Lentivirus carrying FGF2 gene and GFP tag was employed to infect RAW 264.7 cells. Macrophages cell line with FGF2 overexpression (OE) was successfully constructed. The green fluorescence in macrophages post-transfection was observed under fluorescence microscope (Fig 1A). RT-qPCR results showed that FGF2 gene was significantly elevated (Fig 1C). ELISA was used to detect the content of FGF2 in macrophages conditioned medium after transfection, and the results indicated significant differences in the overexpression of FGF2 (Fig 1B).

We also utilized red fluorescent protein (RFP) to label FGF2 in RAW 264.7 cells, and it was observed that the red fluorescence in FGF2 OE group was significantly higher than that in control group. These results suggested that FGF2 OE macrophage cell lines were successfully established (Fig 1D).

### Proteomic analysis of conditioned medium

**Fig 2:**
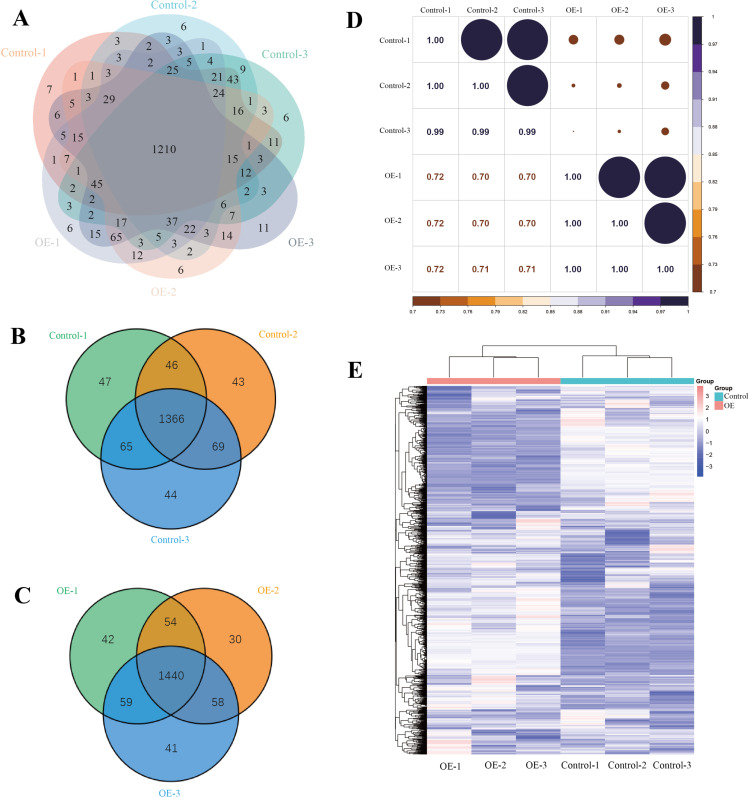
Results of protein quantitative analysis by proteomic analysis. A) Venn diagram of protein quantity analysis results of FGF2 OE group and control group. B) Venn diagram of protein quantity analysis results of FGF2 OE group. C) Venn diagram of protein quantity analysis results of control group. D) Pearson correlation coefficients of all quantitative protein expression between each two samples. E) Cluster heat map of the variation of the study sample.

To confirm whether macrophages conditioned medium contains material basis for wound healing, we performed proteomic analysis using label-free technology. By detecting the protein expression profiles in macrophages conditioned medium (control group) and conditioned medium of FGF2 overexpressed macrophages (OE group), a total of 1842 proteins and 18004 peptides were identified. Venn diagram showed that there were 1440 common proteins in the overexpressed group (Fig 2C) and 1366 common proteins in the control group (Fig 2B). 1210 common proteins were identified between the OE group and the control group (Fig 2A). Detailed quality control analysis was shown in S1 Fig.

In order to reflect the correlation of protein expression between samples, Pearson correlation coefficients of all quantitative protein expression between each pair of samples were calculated separately, and these coefficients were reflected in the form of a matrix figure (Fig 2D). According to the distribution of experimental data of all identified proteins, we drew a cluster heat map to directly reflect the variation of the study sample (Fig 2E). It could be seen that the correlation within the OE groups was high, as was the case in the control groups. There were significant differences between the OE groups and the control groups.

Then we focused on the screening and analysis of differential proteins. To ascertain the statistical significance of the differences, the quantitative values of each protein in two compared sample groups were examined by T-test.

**Fig 3:**
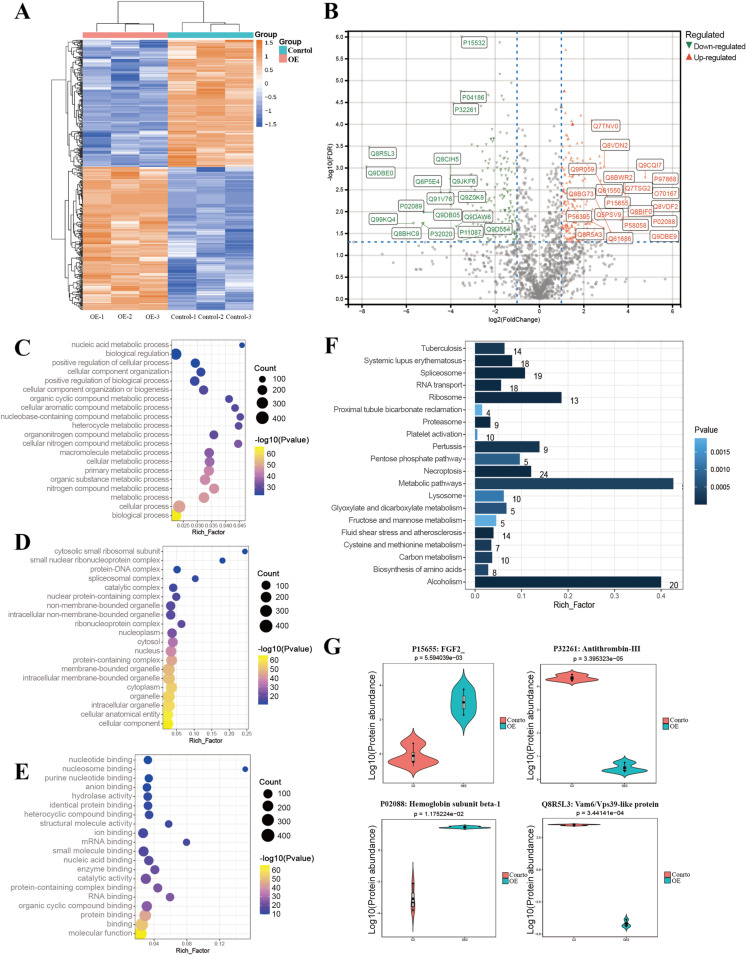
Proteomic analysis of differential proteins. A) Cluster heat map of the differential proteins. B) Volcano plot depicting differential proteins between OE groups and control groups. C) GO analysis of differential proteins (result of BP). D) GO analysis of differential proteins (result of CC). E) GO analysis of differential proteins (result of CC). F) KEGG analysis of differential proteins. G) Violin diagram of important differential proteins. BP: Biological Process, CC: Cellular Component, MF: Molecular Function, GO: Gene Ontology, KEGG: Kyoto Encyclopedia of Genes and Genomes.

The outcomes revealed that 442 proteins were differentially expressed between the control group and the OE group (Fig 3A). Among them, 234 proteins in the OE group were significantly higher than those in control group, while 208 proteins manifested lower expression than in control group (Fig 3B). Subsequently, we bioinformatically explored the biological processes in which they were involved. We performed Gene Ontology (GO) and Kyoto Encyclopedia of Genes and Genomes (KEGG) enrichment analysis for differential proteins. GO analysis showed that differential proteins were mainly concentrated in the biological processes related to cell metabolism (Fig 3C,D,E). KEGG analysis also indicated that differential proteins were concentrated in metabolism-related pathways (Fig 3F).

In conclusion, in addition to the biological effects caused by the differential expression of FGF2 protein, other differential protein-related metabolic pathways are likely to affect wound healing. By analyzing the significance and biological function of these differential proteins, we discovered that some differential proteins such as Hemoglobin subunit beta-1, antithrombin-III and Vam6/Vps39-like protein were changed (Fig 3G).

### Analysis of hyaluronic acid microneedles

**Fig 4:**
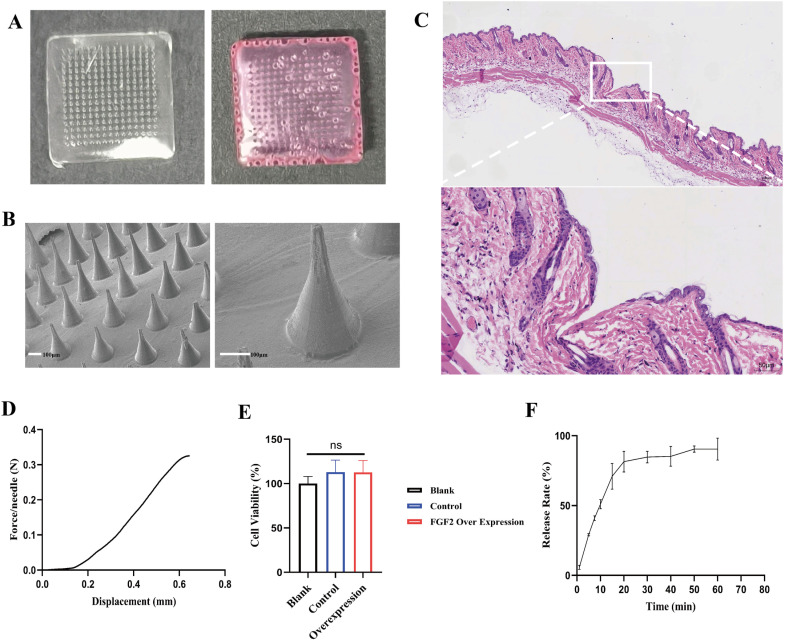
Preparation and characterization of hyaluronic acid microneedles. A) Blank hyaluronic acid microneedles were transparent, while those filled with conditioned medium were red. B) The morphology of microneedles was observed via scanning electron microscope (scale bar: 100 μm). C) Histochemical results of microneedles indentation in tissue. D) Mechanical property test of hyaluronic acid microneedles. E) Cytocompatibility test of the microneedles. F) Dissolution and release property of hyaluronic acid microneedles. N: Newton.

Hyaluronic acid microneedles were prepared by vacuum negative pressure defoaming using PDMS microneedle mold. Blank hyaluronic acid microneedles are transparent, whereas those filled with conditioned medium are red (Fig 4A). The prepared hyaluronic acid microneedles patch was in the shape of a square with a side length of 2 cm, and the number of microneedles in each patch was 15 ×  15. Each microneedle was approximately 250 μm in length, and its base was circular with a diameter of 150 μm (Fig 4B).

Mechanical property test *in vitro*: The mechanical stress of the prepared microneedles was examined using a texture analyzer. The microneedles patch was placed horizontally on the platform with the needle tip upward. The probe compressed the microneedles vertically at a speed of 0.1 mm/s, and the compression force was recorded. Fig 4D showed the relationship between mechanical force and displacement that each microneedle could endure. The mechanical force required for microneedles to penetrate the skin should be greater than 0.045 N. The stress of the microneedles obtained in this study was greater than 0.32 N when the compression displacement reaches 600 μm. This indicated that the obtained microneedles had excellent mechanical properties and could penetrate the skin smoothly.

Dissolution and release property *in vitro*: The drug release concentration was obtained from measured data and standard curve. As shown in Fig 4F, most of the drugs had been released at 60 min, which indicated that the microneedles could release the contained drugs rapidly.

Cytocompatibility test: Cell Counting Kit-8 (CCK-8) Assay Kit was used to assay cytotoxicity *in vitro*. As shown in Fig 4E, the microneedles demonstrated non-toxicity and excellent cytocompatibility.

Microneedle indentation test: The results of the microneedle indentation experiment are shown in Fig 4C. The epidermal tissue was severed after the microneedles penetrated the skin. They entered the tissue at a depth of approximately 200–250 μm. The tip of the microneedles could penetrate the entire layer of the skin.

### Cell scratch assay

**Fig 5:**
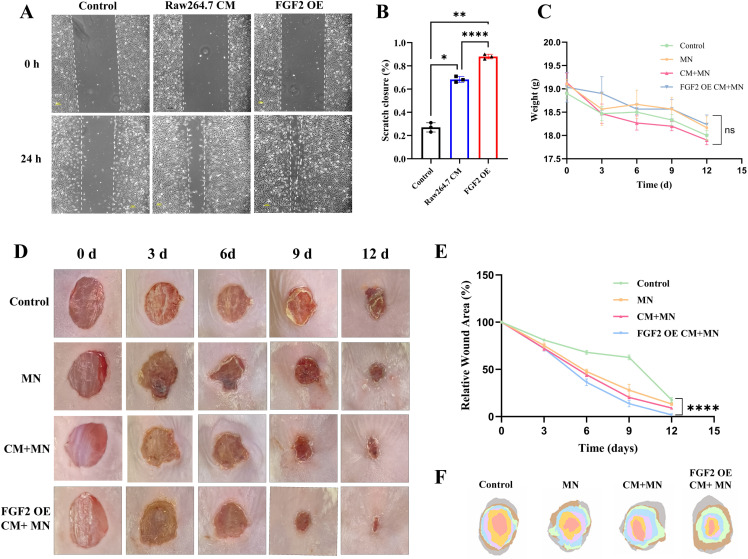
Macrophages conditioned medium promoted wound healing *in vitro* and *in vivo.* A) Representative images of the cell scratch assay of EA.hy 926 cells treated with PBS (control group), macrophages conditioned medium (RAW 264.7 CM group) and conditioned medium of FGF2 overexpressed macrophages (FGF2 OE group) for 24 hours. B) Quantitative analysis of scratch closure evaluated via cell scratch assay. C) The body weight change in each group. D) Photographs of mouse wounds at different time points following various treatments. E) Statistical analysis of the wound areas. F) Simulated *in vivo* wound closure traces for different treatments. CM: Conditioned medium, MN: Microneedles, OE: Overexpression, * p <  0.05, **p <  0.01, ****p <  0.0001.

The cell scratch assay demonstrated that EA.hy 926 cells treated with the conditioned medium of FGF2 overexpressed macrophages (FGF2 OE group) and macrophages conditioned medium (RAW 264.7 CM group) exhibited markedly enhanced migration abilities at 24 hours (Fig 5A). Among them, the FGF2 OE group possessed the strongest ability to enhance cell migration (Fig 5B).

### Effect of the microneedles on wound healing in diabetic mouse models

The wound treatment procedures were displayed in Fig 5D and Fig 5F: A full-thickness wound model of diabetic mice was established, followed by treatment with control, MN, CM +  MN, or FGF2 OE CM +  MN. Macroscopically, the wounds in the FGF2 OE CM +  MN group healed fastest, with almost complete closure of the wound and the formation of new skin on day 12. The wound healing speed of MN group and CM +  MN group were also better than that of control group, but lower than that of the FGF2 OE CM +  MN group (Fig 5E). These ﬁndings demonstrated that culture medium of FGF2 overexpressed macrophages combined with microneedles (FGF2 OE CM +  MN) could considerably improving diabetic wound healing. At the same time, there was no significant difference in body weight among all groups (Fig 5C).

### Histological and immunohistochemical analysis of regenerated skin tissue

**Fig 6:**
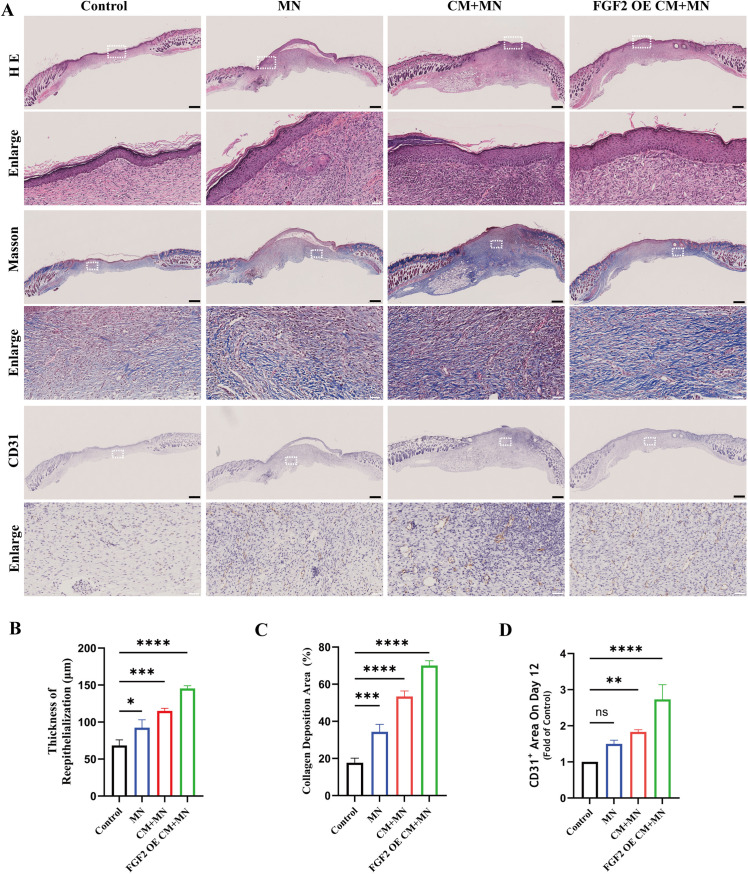
Histological and immunohistochemistry analysis of regenerated skin tissue. A) H&E and Masson staining of the collected wound skin tissue in different groups on day 12. Immunohistochemical staining was employed to show platelet endothelial cell adhesion molecule-1 (CD31). B) The thickness of re-epithelialization on day 12. C) Collagen deposition of wound on day 12. D) Statistical analysis of the CD31^ +^ at the wounds on day 12. * p <  0.05, **p <  0.01, ****p <  0.0001; ns not significant, black scale bar =  500 μm, white scale bar =  50 μm.

Hematoxylin-eosin (H&E) staining and Masson staining were performed to investigate the histological changes in wounds. As shown in Fig 6A, it could be observed that the thickness of reepithelization in FGF2 OE CM +  MN group was thicker than that in other groups on day12 (Fig 6B). Masson staining revealed that the FGF2 OE CM +  MN group displayed more collagen deposition, with the collagen ﬁbers in skin tissues being denser, thicker and better-arranged. Conversely, the control group presented only a small number of collagen ﬁbers that were loose and disordered. The results of MN group and CM +  MN group were between the above two groups (Fig 6C). These results demonstrated that the FGF2 OE CM +  MN group facilitated wound healing by promoting reepithelization and collagen deposition in the diabetic mouse wound.

To understand the underlying mechanisms behind the promotion effects of FGF2 OE CM +  MN on reepithelization during proliferative phase, we investigated the vascularization in wound tissues. As shown in Fig 6D, CD31^ +^ cells were more widely present in the FGF2 OE CM +  MN group. This indicated that the FGF2 OE CM +  MN could more effectively stimulate angiogenesis in proliferative stage.

FGF2 (basic fibroblast growth factor; bFGF) is of crucial significance for effective wound healing [19]. Researches have shown that the localized use of FGF2 is a promising approach for enhancing tissue regeneration, particularly in cases of chronic wounds that exhibit poor healing capabilities or are associated with an abnormal healing process [20–23]. Researchers have developed a variety of different ways to preserve the efficacy of FGF2. In this study, the FGF2 gene was transfected into RAW 264.7 cells by lentivirus. The macrophage cell line with FGF2 overexpression were successfully prepared. Then, the macrophage conditioned medium was collected for proteomic analysis. We found that in addition to FGF2 overexpression, the activation of many cellular metabolic pathways might promote wound repair. Among them, the improvement of vascularization level is particularly important. Subsequently, the microneedle patches were prepared by embedding macrophages conditioned medium in hyaluronic acid microneedles. The microneedles effectively promoted wound healing in diabetic mice *in vivo*. On the one hand, these findings confirmed that macrophages conditioned medium could promote the healing of diabetic wounds, especially conditioned medium of FGF2 overexpressed macrophages. On the other hand, this study also provided an effective way for the administration of macrophages conditioned medium on the wound.

Diabetic wounds are characterized by persistent inflammatory response, inadequate tissue blood supply, local malnutrition and slow healing [24]. The down-regulated expression of extracellular matrix components in diabetic wound impaired the mobile scaffold of tissue repair cells and delayed the migration of repair cells and wound healing [25,26]. The main tissue repair cells, especially fibroblasts and vascular endothelial cells, exhibited decreased proliferation or increased apoptosis, which further reduced the ability of wound repair [27–29]. The expression of various growth factors in diabetic wounds is reduced, and their activities may also be compromised. The same was true for growth factor receptors [30]. All these will impact the regulatory function of the signal transduction pathway of cell proliferation and migration. The function of inflammatory cells was also significantly aberrant. For example, the secretory function and phagocytosis of macrophages were impaired [31,32]. The result was a prolonged period of wound inflammation that could not transition to the stage of vascular granulation proliferation, which also affected wound healing.

Proteomic analysis of conditioned medium of FGF2 overexpressed macrophages revealed significant changes in various cellular metabolic pathways related to the aforementioned functions. For example, as presented in the results, metabolic process, nitrogen compound metabolic process, organic substance metabolic process, primary metabolic process, cellular metabolic process, macromolecule metabolic process, heterocycle metabolic process and so on were all affected. FGF2 is an important angiogenic stimulating factor and the strongest known mitogen. FGF2 induces migration and chemotaxis of vascular endothelial cells, which has a powerful function of promoting angiogenesis [12]. It is an important cytokine for improving the therapeutic effect of chronic ischemic wounds. According to our study, overexpression of FGF2 in macrophages promoted diabetic wound healing. At the same time, some proteins related to vascularization in the cellular pathway may also play an important role. These proteins may exert a synergistic effect with FGF2 to promote the healing of diabetic wounds. For example, proteomic results suggested that Hemoglobin subunit beta-1 which is involved in oxygen transport from the lung to the various peripheral tissues was elevated in the conditional medium of the FGF2 OE group (Fig 3G). This led to an increase in the oxygen-carrying capacity of hemoglobin, thereby enhancing the oxygen supply to the wound. The change of antithrombin-III in conditioned medium may affect the coagulation process and healing speed of wounds. It is the most important serine protease inhibitor in plasma that regulates the blood coagulation cascade. In addition to protein changes associated with vascularization levels, proteins that affected tissue autophagy levels were also noteworthy [33]. For example, the Vam6/Vps39-like protein shown in Fig 3G was associated with autophagy [34]. These findings also provided valuable research targets for subsequent researches.

Most of the previous studies concentrated on the effect of macrophage polarization on wound healing, especially M2 polarization [35,36]. Meanwhile, some researches showed that conditioned medium of macrophage might promote tissue regeneration [8,10,11]. The effect of macrophages conditioned medium on diabetic wound has not been reported. Through this study, it was shown that macrophages conditioned medium, especially FGF2 overexpression group, could indeed promote the healing of diabetic wounds. Overexpression of FGF2 in macrophages further improved its ability to promote wound healing. This study innovatively explored the effect of engineering macrophages biomaterials on diabetic wounds. These works provided a new strategy for diabetic wound repair through the concept of “engineering macrophages as the pharmaceutical factories”.

Biomaterials have emerged as a promising strategy in the process of tissue repair [37,38]. Microneedles, as a rapid and direct approach for drug delivery, have received more and more attention and research in recent years [39]. They enhance the efficiency and permeability of transdermal drug delivery by penetrating the skin barrier and delivering drugs to the wound bed. Hyaluronic acid microneedles possess advantages such as excellent biocompatibility, non-toxicity, biodegradability, non-immunogenicity, water solubility and high molecular compatibility with drugs [15]. Despite the obvious advantages of microneedles, there are also some challenges, such as insufficient penetration, fragility and so on. The microneedle patch was prepared by combining the macrophages conditioned medium with hyaluronic acid microneedles, which could be directly inserted into the depth of skin and wound to promote healing. At the same time, these microneedles prepared by us also have good mechanical properties and biocompatibility. We demonstrated its strong pro-healing effect in diabetic mouse wound model. Immunohistochemical results revealed that the vascularization level of the wound was significantly improved after treatment. This was consistent with the conclusion of proteomics.

This study innovatively demonstrated that engineered macrophage hyaluronic acid microneedles could improve tissue vascularization and promote wound healing in diabetic wounds. It is a new and effective method for the treatment of diabetic wounds. Since the conditioned medium of macrophages culture medium does not involve any ethical issues, and is accessible and conducive to mass production. Consequently, it is highly probable to possess extremely extensive clinical applications in the future

Limitations: This study has not investigated the effect of conditioned medium of macrophages with different polarization states on diabetic wound healing. It was also worth noting that some key proteins that promote wound healing in macrophages conditioned medium need to be further explored and verified. These proteins have the potential to become new targets for wound treatments. All of these need to be further studied and improved by follow-up experiments.

## Conclusion

In summary, we developed hyaluronic acid microneedles loaded with conditioned medium of FGF2 overexpressed macrophages. The microneedles have been proved to enhance tissue vascularization and promote diabetic wound healing. Through diverse modification of macrophages (engineering macrophages) and the combination of appropriate carrier materials, it is likely to become a highly promising therapeutic modality for diabetic wounds.

## Supporting information

S1 Fig Detailed quality control analysis of proteomics. (TIF)

S1 File Minimal data set. (ZIP)

Scheme 1 Schematic diagram of the diabetic wound healing promoting by conditioned medium of FGF2 overexpressed macrophages (engineering macrophages) combined with soluble microneedles. (TIF)
