# Supplementary figures and images for: Conditioned medium of engineering macrophages combined with soluble microneedles promote diabetic wound healing
Source: PLoS One. 2025 Mar 12;20(3):e0316398. doi: 10.1371/journal.pone.0316398 (PMC11902060; doi:10.1371/journal.pone.0316398)

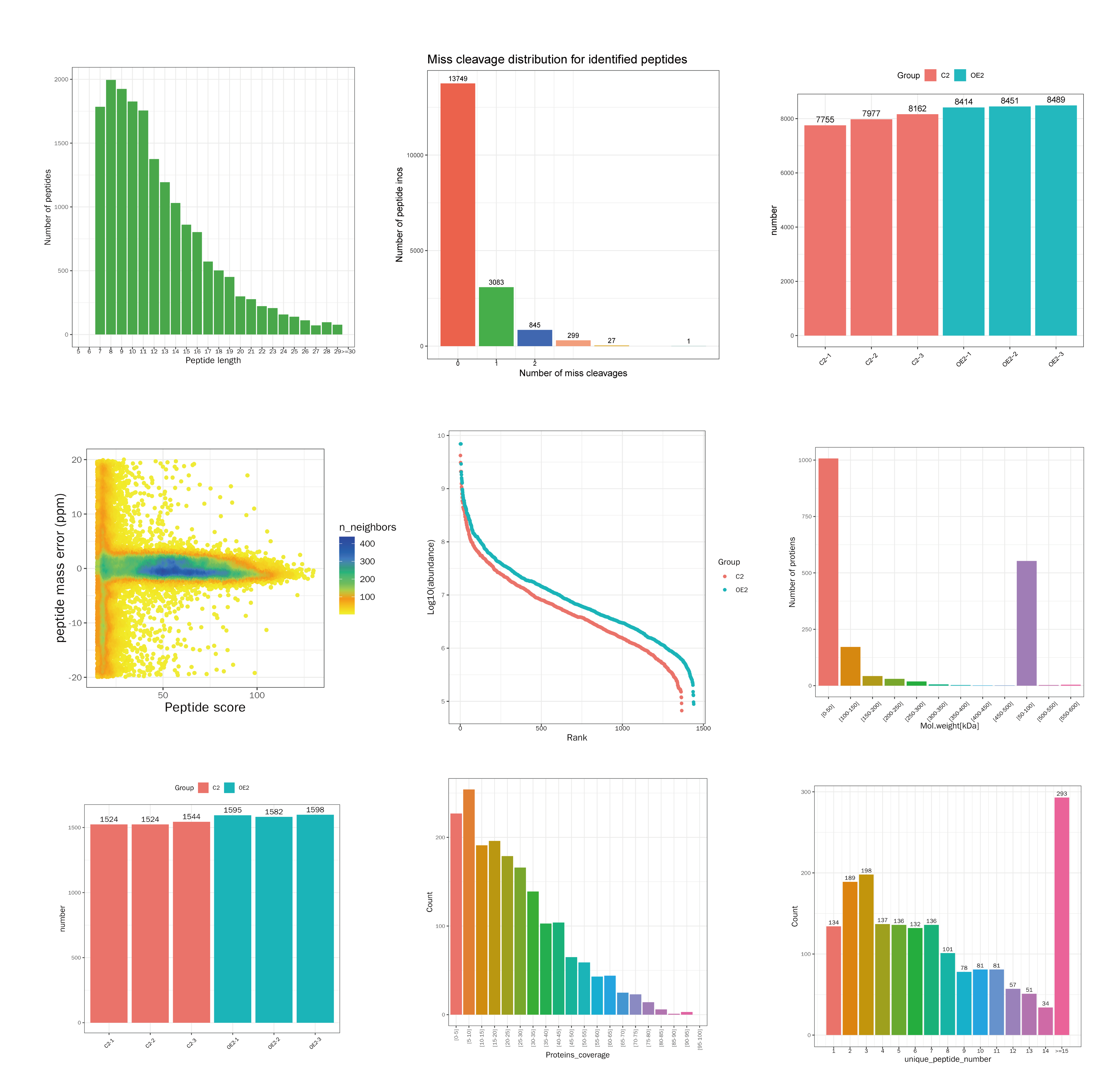

Supplement: S1 Fig — (TIF) [file pone.0316398.s001.tif]

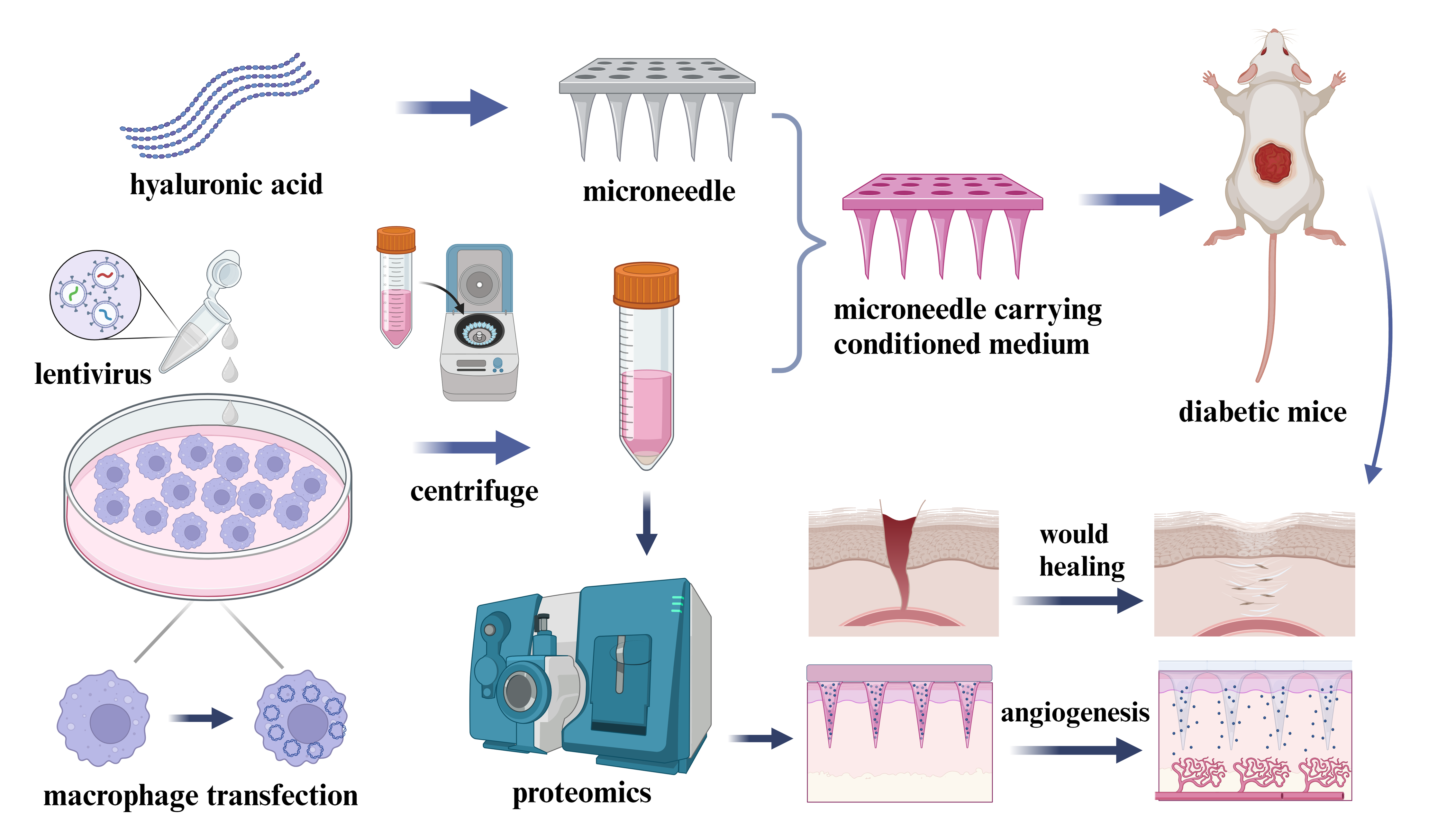

Supplement: Scheme 1 — (TIF) [file pone.0316398.s003.tif]
