# Supplementary material for: Conditioned medium of engineering macrophages combined with soluble microneedles promote diabetic wound healing
Source: PLoS One. 2025 Mar 12;20(3):e0316398. doi: 10.1371/journal.pone.0316398 (PMC11902060; doi:10.1371/journal.pone.0316398)
Supplement: S1 File — (ZIP) [file pone.0316398.s002.zip › S1 File/data/result.docx]

Concentration of FGF2

| Control | FGF2 OE |
| --- | --- |
| 91.5423 | 279.081948 |
| 116.9744 | 253.068972 |
| 107.5151 | 297.751548 |

Relative mRNA level adjusted to ACTIN

| Control | FGF2 OE |
| --- | --- |
| 7.861612328 | 493.182734 |
| 0.357123087 | 376.5787641 |
| 0.356180754 | 448.3538364 |

CCK8

| Blank | Blank | Blank | Control | Control | Control | Overexpression | Overexpression | Overexpression |
| --- | --- | --- | --- | --- | --- | --- | --- | --- |
| 93.41825902 | 97.66454352 | 108.9171975 | 108.7048832 | 128.0254777 | 101.910828 | 128.0254777 | 106.1571125 | 104.0339703 |

Cell scratch assay

| Control | Raw264.7 CM | FGF2 OE |
| --- | --- | --- |
| 0.23 | 0.71 | 0.9 |
| 0.31 | 0.68 | 0.88 |
| 0.27 | 0.66 | 0.86 |
